# Adapting Nutrition Programming for Intergenerational Implementation

**DOI:** 10.1093/geroni/igab046.188

**Published:** 2021-12-17

**Authors:** Shannon Jarrott, Rachel Scrivano, Jill Juris Naar, Alicia Bunger

**Affiliations:** 1 The Ohio State University, Columbus, Ohio, United States; 2 Appalachian State University, Boone, North Carolina, United States

## Abstract

Practitioners frequently tailor programming to meet participant characteristics and logistic constraints, or to incorporate diverse participants, such as intergenerational programming. Adapted programming may be responsive but reduce impact on outcomes. With growing interest in and limited availability of intergenerational protocol, implementation science guides program tailoring to ensure that youth and older adults mutually benefit from adapted programming. We integrated guidelines for tailoring interventions (Framework for Reporting Adaptations and Modifications-Expanded: FRAME) and evidence-based intergenerational practice. We illustrate how program fidelity can be supported in intergenerational settings using examples from an adapted USDA-approved preschool nutrition curriculum delivered intergenerationally. Program acceptability, appropriateness, and feasibility were rated favorably by program stakeholders, and observational implementation data suggest fidelity can be maintained using evidence-based intergenerational strategies. Our findings support the potential for protocol developed for one age group to benefit youth and older adults when it is adapted using implementation and intergenerational guidelines.

